# (*E*)-(25*S*)-23-Acetyl-5β-furost-22-ene-3β,26-diol

**DOI:** 10.1107/S1600536808004509

**Published:** 2008-02-22

**Authors:** María-Guadalupe Hernández Linares, Jesús Sandoval Ramírez, Socorro Meza Reyes, Sara Montiel Smith, Sylvain Bernès

**Affiliations:** a Escuela de Ingeniería Química, Universidad del Istmo, Ciudad Universitaria S/N, 70760 Sto. Domingo Tehuantepec, Oax., Mexico; b Facultad de Ciencias Químicas, Benemérita Universidad Autónoma de Puebla, Ciudad Universitaria, San Manuel, 72000 Puebla, Pue., Mexico; c DEP Facultad de Ciencias Químicas, UANL, Guerrero y Progreso S/N, Col. Treviño, 64570 Monterrey, NL, Mexico

## Abstract

The title steroid, C_29_H_46_O_4_, is a furostene derivative with a C=C double-bond length of 1.353 (3) Å and an *E* configuration. The side chain is oriented toward the α face of the *A*–*E* steroidal nucleus and presents a disordered terminal CH_2_—OH group [occupancies for resolved sites are 0.591 (9) and 0.409 (9)]. The methyl group at C20 attached to ring *E* is also oriented toward the α face, avoiding steric hindrance with the carbonyl O atom of the acetyl group. The furostene and acetyl functionalities form an α,β-unsaturated ketone system, with an *s*-*cis* configuration. All hydr­oxy and carbonyl groups are involved in weak inter­molecular hydrogen bonds. The absolute configuration was assigned from the synthesis.

## Related literature

The diacetate of the title compound has been characterized by X-ray crystallography (Sandoval-Ramírez *et al.*, 2003 ▶), as well as a related furost-22-ene derivate with the C20 site substituted by a methyl group and an acetyl group (Meza-Reyes *et al.*, 2004 ▶).
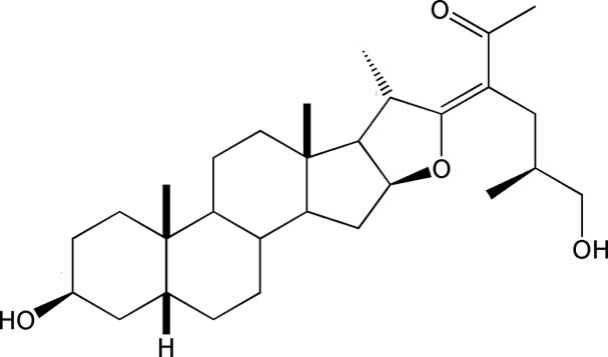


## Experimental

### 

#### Crystal data

C_29_H_46_O_4_
*M* *_r_* = 458.66
Monoclinic, 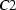

*a* = 23.568 (2) Å
*b* = 7.8420 (9) Å
*c* = 14.7840 (14) Å
β = 101.046 (5)°
*V* = 2681.7 (5) Å^3^
*Z* = 4
Mo *K*α radiation
μ = 0.07 mm^−1^
*T* = 296 (1) K
0.65 × 0.35 × 0.32 mm

#### Data collection

Bruker *P*4 diffractometer
Absorption correction: none
4351 measured reflections
2559 independent reflections
2121 reflections with *I* > 2σ(*I*)
*R*_int_ = 0.026
3 standard reflections every 97 reflections intensity decay: 1%

#### Refinement

*R*[*F* ^2^ > 2σ(*F* ^2^)] = 0.038
*wR*(*F* ^2^) = 0.097
*S* = 1.02
2559 reflections
323 parameters
2 restraints
H-atom parameters constrained
Δρ_max_ = 0.13 e Å^−3^
Δρ_min_ = −0.13 e Å^−3^

### 

Data collection: *XSCANS* (Siemens, 1996 ▶); cell refinement: *XSCANS*; data reduction: *XSCANS*; program(s) used to solve structure: *SHELXTL-Plus* (Release 5.10; Sheldrick, 2008 ▶); program(s) used to refine structure: *SHELXTL-Plus*; mol­ecular graphics: *SHELXTL-Plus*; software used to prepare material for publication: *SHELXTL-Plus*.

## Supplementary Material

Crystal structure: contains datablocks I, global. DOI: 10.1107/S1600536808004509/rz2197sup1.cif

Structure factors: contains datablocks I. DOI: 10.1107/S1600536808004509/rz2197Isup2.hkl

Additional supplementary materials:  crystallographic information; 3D view; checkCIF report

## Figures and Tables

**Table 1:** Selected torsion angles (°)

C20—C22—C23—C23′	−3.5 (4)
C20—C22—C23—C24	175.4 (3)
O22′—C22—C23—C23′	179.3 (2)
O22′—C22—C23—C24	−1.8 (4)

**Table 2:** Hydrogen-bond geometry (Å, °)

*D*—H⋯*A*	*D*—H	H⋯*A*	*D*⋯*A*	*D*—H⋯*A*
O3′—H3′⋯O23"^i^	0.98	1.89	2.841 (3)	163
O26*A*—H26*E*⋯O3′^ii^	0.82	2.20	2.951 (6)	153
O26*B*—H26*F*⋯O3′^ii^	0.82	2.24	2.919 (16)	140

Symmetry codes: (i) 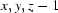; (ii) 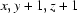.

**Fig. 1.**
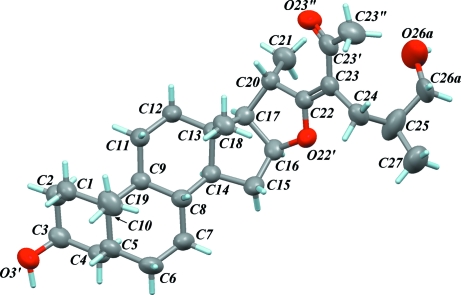
The molecular structure of the title compound with 50% displacement ellipsoids for non-H atoms. Disordered atoms C26b and O26b (lateral chain) have been omitted for clarity.

## supplementary crystallographic information

### Comment

We are interested in the preparation of new steroidal derivatives, through the cleavage of the *F* ring in sarsasapogenin (Sandoval-Ramírez *et al.*, 2003; Meza-Reyes *et al.*, 2004). Such reactions are valuable entries to furostenes; the title compound is a new representative of this family.

The molecular conformation of the title compound compares well with that previously observed for the corresponding diacetate (Sandoval-Ramírez *et al.*, 2003). The functionality C22?C23 has a bond length of 1.353 (3) Å [1.355 (3) Å for the diacetate] and is *E* configured. The side chain C24/C25/C26/O26 is oriented toward the *α* face of the *A*–*E* steroidal nucleus (Fig. 1). The terminal group CH_2_—OH is disordered over two positions (denoted a and b), and has a poorly defined geometry (see *Experimental*). The acetyl group substituting the furostenic atom C23 forms a *s*-*cis α*,*β*-unsaturated ketone system with the *E* configuration at the C22?C23 double bond. The methyl group C21 is placed on the *α* steroidal face, in agreement with a general rule followed by furostenes: the bulkier group at C20 avoids steric hindrance with groups substituting C23 (Meza-Reyes *et al.*, 2004). The solid-state conformation of the title compound is retained in solution, as confirmed by NMR data.

The crystal structure contains rather weak intermolecular hydrogen bonds, involving all hydroxyl (O3', O26*a*/O26*b*) and carbonyl (O23") groups. The main contact, O3'—H3'···O23", links molecules into chains running in the [001] direction.

### Experimental

(*E*)-(25*S*)-23-Acetyl-3*β*,26-diacetoxy-5*β*,16*β*-furost-22-ene (500 mg, 0.9 mmol) was added under stirring to a 10% ethanolic solution of KOH (25 ml), following the reaction course by TLC. After completion, the mixture was treated with saturated NaCl and washed with water. The organic phase was extracted using CH_2_Cl_2_ (3×30 ml) and then dried over Na_2_SO_4_. After removing solvent, the crude was chromatographed (ethyl acetate/hexane, 2:3). Single crystals were obtained from an ethyl acetate solution, at 298 K. (I) has been characterized by spectroscopy: ^1^H-NMR, δ (400 MHz, CDCl_3_, 298 K) 4.10 (1*H*, s, H-3), 4.96 (1*H*, ddd, *J* = 4.2, 7.6 and 11.6 Hz, H-16), 1.86 (1*H*, d, *J* = 7.3 Hz, H-17), 0.60 (3*H*, s, H-18), 0.95 (3*H*, s, H-19), 3.70 (1*H*, c, *J*_20–21_ = 7.0 Hz, H-20), 2.24 (3*H*, s, H-23^2^), 1.27 (3*H*, d, *J*_21–20_ = 7.0 Hz, H-21), 2.44 and 2.22 (2*H*, ABX system, H-24), 3.38 and 3.45 (2*H*, ABX system, H-26), 0.94 (3*H*, d, *J*_27–25_ = 7.0 Hz, H-27). ^13^C-NMR, δ (100 MHz, CDCl_3_, 298 K) 29.87 (C-1), 27.78 (C-2), 66.95 (C-3), 30.38 (C-4), 36.40 (C-5), 26.38 (C-6), 26.44 (C-7), 35.77 (C-8), 39.90 (C-9), 35.22 (C-10), 20.39 (C-11), 38.46 (C-12), 41.75 (C-13), 55.52 (C-14), 34.98 (C-15), 86.29 (C-16), 62.59 (C-17), 13.35 (C-18), 23.81 (C-19), 37.84 (C-20), 20.17 (C-21), 178.60 (C-22), 108.32 (C-23), 33.43 (C-24), 30.27 (C-25), 66.56 (C-26), 17.08 (C-27), 199.08 (C-23^1^), 29.24 (C-23^2^). IR (KBr, ν_max_, cm^-1^): 3498 (OH), 1653 (*α*,*β*-unsaturated C?O). [α]^20^_D_ = +119.17 (*c* = 0.01 g.ml^-1^, CHCl_3_).

### Refinement

The final part of the lateral chain is badly disordered. Atoms C26 and O26 were modeled over two sites, and occupancies refined with the sum for a single atom constrained to 1. Site occupation factors converged to 0.591 (9) (C26*a* and O26*a*) and 0.409 (9) (C26*b* and O26*b*). Bond length C25—C26*a* was restrained to 1.50 (1) Å, while other dimensions were refined freely. The poor geometry for this part probably indicates that the actual disorder is more complex than a two-sites model. C-bonded H atoms were placed in idealized positions and refined as riding to their parent atoms. C—H bond lengths were fixed to 0.98 (methine CH), 0.97 (methylene CH_2_) or 0.96 Å (methyl CH_3_) and isotropic displacement parameters calculated as *U*_iso_(H) = *xU*_eq_(carrier C) with *x* = 1.5 (CH_3_) or *x* = 1.2 (CH_2_ and CH). Disordered hydroxyl H atoms H26E and H26F were placed in idealized positions and refined fixing O—H bond lengths to 0.82 Å and *U*_iso_(H) = 1.5*U*_eq_(carrier O). Finally, hydroxyl H atom H3' was found in a difference map and refined with this as-found position and *U*_iso_(H3') = 1.5*U*_eq_(O3'). Measured Friedel pairs (1496) were merged for refinement.

### Crystal data

**Table:**

C_29_H_46_O_4_	*D*_x_ = 1.136 Mg m^−^^3^
*M**_r_* = 458.66	Melting point = 354–356 K
Monoclinic, *C*2	Mo *K*α radiation λ = 0.71073 Å
Hall symbol: C 2y	Cell parameters from 74 reflections
*a* = 23.568 (2) Å	θ = 4.9–12.2º
*b* = 7.8420 (9) Å	µ = 0.07 mm^−^^1^
*c* = 14.7840 (14) Å	*T* = 296 (1) K
β = 101.046 (5)º	Cell measurement pressure: 101(2) kPa
*V* = 2681.7 (5) Å^3^	Irregular, colourless
*Z* = 4	0.65 × 0.35 × 0.32 mm
*F*_000_ = 1008	

### Data collection

**Table:**

Bruker P4 diffractometer	*R*_int_ = 0.026
Radiation source: fine-focus sealed tube	θ_max_ = 25.0º
Monochromator: graphite	θ_min_ = 2.0º
*T* = 296(1) K	*h* = −1→28
*P* = 101(2) kPa	*k* = −9→5
2θ/ω scans	*l* = −17→17
Absorption correction: none	3 standard reflections
4351 measured reflections	every 97 reflections
2559 independent reflections	intensity decay: 1%
2121 reflections with *I* > 2σ(*I*)	

### Refinement

**Table:**

Refinement on *F*^2^	Secondary atom site location: difference Fourier map
Least-squares matrix: full	Hydrogen site location: inferred from neighbouring sites
*R*[*F*^2^ > 2σ(*F*^2^)] = 0.039	H-atom parameters constrained
*wR*(*F*^2^) = 0.097	*w* = 1/[σ^2^(*F*_o_^2^) + (0.0494*P*)^2^ + 0.4384*P*] where *P* = (*F*_o_^2^ + 2*F*_c_^2^)/3
*S* = 1.02	(Δ/σ)_max_ < 0.001
2559 reflections	Δρ_max_ = 0.13 e Å^−^^3^
323 parameters	Δρ_min_ = −0.13 e Å^−^^3^
2 restraints	Extinction correction: SHELXTL-Plus, Fc^*^=kFc[1+0.001xFc^2^λ^3^/sin(2θ)]^-1/4^
Primary atom site location: structure-invariant direct methods	Extinction coefficient: 0.0026 (5)

### Fractional atomic coordinates and isotropic or equivalent isotropic displacement parameters (Å^2^)

**Table:**

	*x*	*y*	*z*	*U*_iso_*/*U*_eq_	Occ. (<1)
C1	0.34699 (12)	0.0303 (4)	0.50200 (18)	0.0581 (7)	
H1A	0.3696	−0.0244	0.4619	0.070*	
H1B	0.3400	−0.0537	0.5467	0.070*	
C2	0.28939 (12)	0.0842 (5)	0.44463 (18)	0.0638 (8)	
H2A	0.2649	0.1283	0.4851	0.077*	
H2B	0.2703	−0.0147	0.4130	0.077*	
C3	0.29628 (11)	0.2195 (5)	0.37409 (17)	0.0601 (8)	
H3A	0.2580	0.2618	0.3453	0.072*	
O3'	0.32240 (9)	0.1390 (3)	0.30500 (12)	0.0705 (6)	
H3'	0.3269	0.2348	0.2647	0.106*	
C4	0.33188 (11)	0.3665 (4)	0.42067 (17)	0.0558 (7)	
H4A	0.3093	0.4271	0.4587	0.067*	
H4B	0.3394	0.4450	0.3736	0.067*	
C5	0.38998 (11)	0.3145 (4)	0.48114 (16)	0.0544 (7)	
H5A	0.4135	0.2638	0.4402	0.065*	
C6	0.42273 (13)	0.4707 (5)	0.52571 (19)	0.0691 (9)	
H6A	0.4244	0.5557	0.4786	0.083*	
H6B	0.4621	0.4380	0.5524	0.083*	
C7	0.39424 (13)	0.5492 (4)	0.60094 (18)	0.0612 (8)	
H7A	0.4183	0.6413	0.6307	0.073*	
H7B	0.3571	0.5971	0.5728	0.073*	
C8	0.38548 (11)	0.4176 (4)	0.67347 (16)	0.0488 (7)	
H8A	0.4235	0.3826	0.7075	0.059*	
C9	0.35351 (11)	0.2576 (4)	0.62918 (16)	0.0453 (6)	
H9A	0.3155	0.2972	0.5973	0.054*	
C10	0.38278 (10)	0.1769 (4)	0.55348 (16)	0.0495 (7)	
C11	0.34185 (13)	0.1317 (4)	0.70299 (17)	0.0554 (7)	
H11A	0.3183	0.0388	0.6730	0.066*	
H11B	0.3784	0.0837	0.7340	0.066*	
C12	0.31126 (12)	0.2120 (4)	0.77530 (17)	0.0543 (7)	
H12A	0.2722	0.2435	0.7462	0.065*	
H12B	0.3087	0.1283	0.8227	0.065*	
C13	0.34296 (10)	0.3699 (3)	0.82002 (15)	0.0419 (6)	
C14	0.35120 (11)	0.4910 (4)	0.74173 (16)	0.0463 (6)	
H14A	0.3123	0.5116	0.7061	0.056*	
C15	0.36958 (13)	0.6583 (4)	0.79078 (17)	0.0586 (7)	
H15A	0.3614	0.7543	0.7489	0.070*	
H15B	0.4105	0.6577	0.8178	0.070*	
C16	0.33268 (12)	0.6649 (4)	0.86521 (16)	0.0520 (7)	
H16A	0.3018	0.7499	0.8500	0.062*	
C17	0.30738 (10)	0.4861 (4)	0.87245 (16)	0.0446 (6)	
H17A	0.2668	0.4851	0.8410	0.054*	
C18	0.40118 (11)	0.3167 (4)	0.88110 (17)	0.0549 (7)	
H18A	0.4248	0.2613	0.8438	0.082*	
H18B	0.3940	0.2397	0.9281	0.082*	
H18C	0.4208	0.4162	0.9093	0.082*	
C19	0.44245 (12)	0.1014 (5)	0.5966 (2)	0.0722 (9)	
H19A	0.4616	0.0637	0.5484	0.108*	
H19B	0.4373	0.0064	0.6352	0.108*	
H19C	0.4655	0.1871	0.6328	0.108*	
C20	0.31059 (10)	0.4638 (4)	0.97677 (15)	0.0460 (6)	
H20A	0.3199	0.3454	0.9951	0.055*	
C21	0.25366 (14)	0.5186 (5)	1.0048 (2)	0.0666 (8)	
H21A	0.2590	0.5234	1.0708	0.100*	
H21B	0.2239	0.4374	0.9816	0.100*	
H21C	0.2426	0.6291	0.9795	0.100*	
C22	0.35952 (10)	0.5804 (4)	1.01821 (16)	0.0475 (6)	
O22'	0.36797 (8)	0.6985 (3)	0.95547 (11)	0.0601 (5)	
C23	0.39521 (11)	0.5813 (4)	1.10164 (16)	0.0500 (6)	
C23'	0.38752 (13)	0.4580 (4)	1.17199 (17)	0.0554 (7)	
C23"	0.43509 (14)	0.4394 (6)	1.2564 (2)	0.0798 (11)	
H23A	0.4268	0.3440	1.2924	0.120*	
H23B	0.4373	0.5414	1.2928	0.120*	
H23C	0.4714	0.4211	1.2374	0.120*	
O23"	0.34436 (9)	0.3677 (3)	1.16669 (12)	0.0715 (6)	
C24	0.44328 (12)	0.7122 (5)	1.11723 (19)	0.0615 (8)	
H24A	0.4731	0.6725	1.1675	0.074*	
H24B	0.4602	0.7161	1.0624	0.074*	
C25	0.42713 (18)	0.8916 (6)	1.1391 (3)	0.1028 (14)	
H25A	0.3916	0.9267	1.1003	0.123*	0.591 (9)
H25B	0.4080	0.9136	1.0768	0.123*	0.409 (9)
C26A	0.4232 (7)	0.9015 (16)	1.2456 (6)	0.089 (3)	0.591 (9)
H26A	0.4203	1.0204	1.2622	0.107*	0.591 (9)
H26B	0.4589	0.8573	1.2817	0.107*	0.591 (9)
O26A	0.3760 (3)	0.8110 (8)	1.2709 (5)	0.118 (3)	0.591 (9)
H26E	0.3527	0.8792	1.2846	0.177*	0.591 (9)
C26B	0.3808 (5)	0.9329 (14)	1.1735 (7)	0.096 (4)	0.409 (9)
H26C	0.3485	0.8560	1.1544	0.115*	0.409 (9)
H26D	0.3688	1.0503	1.1613	0.115*	0.409 (9)
O26B	0.4123 (8)	0.903 (3)	1.2732 (8)	0.123 (5)	0.409 (9)
H26F	0.3974	0.9613	1.3082	0.184*	0.409 (9)
C27	0.47626 (19)	1.0164 (7)	1.1335 (3)	0.1147 (16)	
H27A	0.4869	1.0071	1.0742	0.172*	
H27B	0.5090	0.9898	1.1807	0.172*	
H27C	0.4637	1.1307	1.1421	0.172*	

### Atomic displacement parameters (Å^2^)

**Table:**

	*U*^11^	*U*^22^	*U*^33^	*U*^12^	*U*^13^	*U*^23^
C1	0.0725 (17)	0.0578 (18)	0.0448 (13)	0.0020 (16)	0.0132 (12)	−0.0001 (13)
C2	0.0620 (15)	0.076 (2)	0.0531 (14)	−0.0184 (16)	0.0093 (12)	−0.0056 (16)
C3	0.0535 (14)	0.078 (2)	0.0470 (13)	−0.0013 (16)	0.0039 (11)	0.0027 (15)
O3'	0.0913 (14)	0.0727 (15)	0.0479 (9)	−0.0049 (13)	0.0146 (9)	0.0015 (11)
C4	0.0602 (15)	0.0618 (19)	0.0464 (13)	0.0021 (16)	0.0126 (12)	0.0106 (14)
C5	0.0495 (14)	0.073 (2)	0.0425 (13)	−0.0035 (14)	0.0132 (11)	0.0026 (14)
C6	0.0679 (17)	0.088 (2)	0.0542 (15)	−0.0252 (19)	0.0196 (13)	0.0086 (18)
C7	0.0704 (17)	0.063 (2)	0.0518 (14)	−0.0228 (16)	0.0146 (13)	0.0034 (14)
C8	0.0441 (12)	0.0570 (18)	0.0441 (12)	−0.0069 (13)	0.0058 (10)	0.0057 (12)
C9	0.0416 (12)	0.0498 (16)	0.0438 (12)	−0.0006 (12)	0.0067 (10)	0.0049 (12)
C10	0.0456 (13)	0.0605 (18)	0.0419 (12)	0.0030 (13)	0.0070 (10)	0.0033 (13)
C11	0.0732 (17)	0.0473 (17)	0.0463 (13)	−0.0074 (15)	0.0131 (12)	0.0016 (13)
C12	0.0686 (16)	0.0490 (16)	0.0480 (13)	−0.0094 (15)	0.0177 (12)	0.0017 (13)
C13	0.0427 (12)	0.0424 (15)	0.0400 (12)	0.0000 (12)	0.0064 (10)	0.0053 (12)
C14	0.0474 (13)	0.0468 (16)	0.0427 (13)	−0.0029 (13)	0.0040 (10)	0.0047 (12)
C15	0.0768 (18)	0.0463 (17)	0.0519 (14)	−0.0076 (15)	0.0105 (13)	0.0070 (14)
C16	0.0603 (15)	0.0444 (16)	0.0478 (13)	0.0051 (13)	0.0018 (11)	0.0027 (13)
C17	0.0389 (12)	0.0488 (16)	0.0429 (12)	0.0007 (12)	−0.0005 (9)	0.0042 (12)
C18	0.0548 (14)	0.0623 (18)	0.0469 (13)	0.0127 (14)	0.0081 (11)	0.0068 (14)
C19	0.0596 (16)	0.098 (3)	0.0585 (15)	0.0196 (19)	0.0089 (13)	−0.0004 (19)
C20	0.0469 (13)	0.0458 (15)	0.0454 (12)	−0.0030 (12)	0.0093 (10)	−0.0009 (12)
C21	0.0573 (14)	0.075 (2)	0.0720 (15)	0.0054 (17)	0.0229 (12)	−0.0050 (17)
C22	0.0550 (14)	0.0441 (14)	0.0453 (12)	0.0000 (13)	0.0142 (11)	−0.0016 (13)
O22'	0.0774 (12)	0.0511 (12)	0.0493 (9)	−0.0155 (11)	0.0058 (8)	0.0015 (9)
C23	0.0502 (13)	0.0565 (17)	0.0439 (12)	−0.0022 (14)	0.0101 (11)	−0.0066 (13)
C23'	0.0585 (16)	0.065 (2)	0.0432 (13)	0.0032 (16)	0.0119 (12)	−0.0062 (14)
C23"	0.0706 (19)	0.109 (3)	0.0560 (16)	0.000 (2)	0.0016 (15)	0.0168 (19)
O23"	0.0800 (13)	0.0824 (16)	0.0506 (10)	−0.0193 (14)	0.0090 (9)	0.0101 (11)
C24	0.0533 (15)	0.083 (2)	0.0492 (13)	−0.0108 (17)	0.0131 (12)	−0.0121 (16)
C25	0.079 (2)	0.067 (2)	0.168 (4)	−0.021 (2)	0.039 (3)	−0.038 (3)
C26A	0.119 (7)	0.087 (5)	0.075 (7)	−0.029 (5)	0.053 (6)	−0.040 (6)
O26A	0.121 (4)	0.106 (4)	0.150 (5)	−0.022 (4)	0.085 (4)	−0.043 (4)
C26B	0.102 (7)	0.080 (7)	0.094 (8)	0.017 (6)	−0.011 (6)	−0.028 (6)
O26B	0.141 (11)	0.182 (12)	0.057 (5)	0.006 (9)	0.047 (6)	−0.008 (6)
C27	0.123 (3)	0.101 (3)	0.127 (3)	−0.054 (3)	0.040 (3)	−0.029 (3)

### Geometric parameters (Å, °)

**Table:**

C1—C2	1.517 (4)	C16—C17	1.536 (4)
C1—C10	1.539 (4)	C16—H16A	0.9800
C1—H1A	0.9700	C17—C20	1.540 (3)
C1—H1B	0.9700	C17—H17A	0.9800
C2—C3	1.518 (4)	C18—H18A	0.9600
C2—H2A	0.9700	C18—H18B	0.9600
C2—H2B	0.9700	C18—H18C	0.9600
C3—O3'	1.436 (3)	C19—H19A	0.9600
C3—C4	1.512 (4)	C19—H19B	0.9600
C3—H3A	0.9800	C19—H19C	0.9600
O3'—H3'	0.9770	C20—C22	1.507 (4)
C4—C5	1.540 (4)	C20—C21	1.539 (4)
C4—H4A	0.9700	C20—H20A	0.9800
C4—H4B	0.9700	C21—H21A	0.9600
C5—C6	1.528 (5)	C21—H21B	0.9600
C5—C10	1.551 (4)	C21—H21C	0.9600
C5—H5A	0.9800	C22—O22'	1.352 (3)
C6—C7	1.534 (4)	C22—C23	1.353 (3)
C6—H6A	0.9700	C23—C23'	1.456 (4)
C6—H6B	0.9700	C23—C24	1.513 (4)
C7—C8	1.531 (4)	C23'—O23"	1.230 (3)
C7—H7A	0.9700	C23'—C23"	1.516 (4)
C7—H7B	0.9700	C23"—H23A	0.9600
C8—C14	1.522 (4)	C23"—H23B	0.9600
C8—C9	1.543 (4)	C23"—H23C	0.9600
C8—H8A	0.9800	C24—C25	1.509 (6)
C9—C11	1.535 (4)	C24—H24A	0.9700
C9—C10	1.557 (3)	C24—H24B	0.9700
C9—H9A	0.9800	C25—C26A	1.597 (7)
C10—C19	1.546 (4)	C25—C26B	1.329 (11)
C11—C12	1.534 (4)	C25—H25A	0.9599
C11—H11A	0.9700	C25—H25B	0.9599
C11—H11B	0.9700	C25—C27	1.531 (6)
C12—C13	1.530 (4)	C26A—O26A	1.428 (15)
C12—H12A	0.9700	C26A—H26A	0.9700
C12—H12B	0.9700	C26A—H26B	0.9700
C13—C14	1.538 (3)	O26A—H26E	0.8200
C13—C17	1.544 (4)	C26B—O26B	1.536 (18)
C13—C18	1.548 (3)	C26B—H26C	0.9700
C14—C15	1.521 (4)	C26B—H26D	0.9700
C14—H14A	0.9800	O26B—H26F	0.8200
C15—C16	1.529 (4)	C27—H27A	0.9600
C15—H15A	0.9700	C27—H27B	0.9600
C15—H15B	0.9700	C27—H27C	0.9600
C16—O22'	1.455 (3)		
			
C2—C1—C10	114.6 (3)	H15A—C15—H15B	109.1
C2—C1—H1A	108.6	O22'—C16—C15	111.2 (2)
C10—C1—H1A	108.6	O22'—C16—C17	105.2 (2)
C2—C1—H1B	108.6	C15—C16—C17	107.5 (2)
C10—C1—H1B	108.6	O22'—C16—H16A	110.9
H1A—C1—H1B	107.6	C15—C16—H16A	110.9
C1—C2—C3	112.1 (2)	C17—C16—H16A	110.9
C1—C2—H2A	109.2	C16—C17—C20	103.2 (2)
C3—C2—H2A	109.2	C16—C17—C13	104.40 (19)
C1—C2—H2B	109.2	C20—C17—C13	120.6 (2)
C3—C2—H2B	109.2	C16—C17—H17A	109.3
H2A—C2—H2B	107.9	C20—C17—H17A	109.3
O3'—C3—C4	112.5 (2)	C13—C17—H17A	109.3
O3'—C3—C2	107.4 (3)	C13—C18—H18A	109.5
C4—C3—C2	110.1 (2)	C13—C18—H18B	109.5
O3'—C3—H3A	108.9	H18A—C18—H18B	109.5
C4—C3—H3A	108.9	C13—C18—H18C	109.5
C2—C3—H3A	108.9	H18A—C18—H18C	109.5
C3—O3'—H3'	102.0	H18B—C18—H18C	109.5
C3—C4—C5	114.6 (3)	C10—C19—H19A	109.5
C3—C4—H4A	108.6	C10—C19—H19B	109.5
C5—C4—H4A	108.6	H19A—C19—H19B	109.5
C3—C4—H4B	108.6	C10—C19—H19C	109.5
C5—C4—H4B	108.6	H19A—C19—H19C	109.5
H4A—C4—H4B	107.6	H19B—C19—H19C	109.5
C6—C5—C4	111.0 (3)	C22—C20—C21	111.1 (2)
C6—C5—C10	111.9 (2)	C22—C20—C17	103.0 (2)
C4—C5—C10	112.4 (2)	C21—C20—C17	111.1 (2)
C6—C5—H5A	107.1	C22—C20—H20A	110.5
C4—C5—H5A	107.1	C21—C20—H20A	110.5
C10—C5—H5A	107.1	C17—C20—H20A	110.5
C5—C6—C7	112.3 (2)	C20—C21—H21A	109.5
C5—C6—H6A	109.1	C20—C21—H21B	109.5
C7—C6—H6A	109.1	H21A—C21—H21B	109.5
C5—C6—H6B	109.1	C20—C21—H21C	109.5
C7—C6—H6B	109.1	H21A—C21—H21C	109.5
H6A—C6—H6B	107.9	H21B—C21—H21C	109.5
C8—C7—C6	111.9 (3)	O22'—C22—C23	118.3 (2)
C8—C7—H7A	109.2	O22'—C22—C20	109.8 (2)
C6—C7—H7A	109.2	C23—C22—C20	131.9 (3)
C8—C7—H7B	109.2	C22—O22'—C16	111.8 (2)
C6—C7—H7B	109.2	C22—C23—C23'	120.5 (3)
H7A—C7—H7B	107.9	C22—C23—C24	117.3 (3)
C14—C8—C7	111.7 (3)	C23'—C23—C24	122.2 (2)
C14—C8—C9	108.12 (19)	O23"—C23'—C23	123.5 (2)
C7—C8—C9	111.8 (2)	O23"—C23'—C23"	118.0 (3)
C14—C8—H8A	108.4	C23—C23'—C23"	118.5 (3)
C7—C8—H8A	108.4	C23'—C23"—H23A	109.5
C9—C8—H8A	108.4	C23'—C23"—H23B	109.5
C11—C9—C8	111.15 (19)	H23A—C23"—H23B	109.5
C11—C9—C10	114.4 (2)	C23'—C23"—H23C	109.5
C8—C9—C10	112.8 (2)	H23A—C23"—H23C	109.5
C11—C9—H9A	105.9	H23B—C23"—H23C	109.5
C8—C9—H9A	105.9	C25—C24—C23	116.9 (2)
C10—C9—H9A	105.9	C25—C24—H24A	108.1
C1—C10—C19	106.6 (3)	C23—C24—H24A	108.1
C1—C10—C5	107.51 (19)	C25—C24—H24B	108.1
C19—C10—C5	109.8 (2)	C23—C24—H24B	108.1
C1—C10—C9	112.5 (2)	H24A—C24—H24B	107.3
C19—C10—C9	110.67 (19)	C26A—C25—H25A	111.5
C5—C10—C9	109.6 (2)	C26B—C25—H25B	93.1
C12—C11—C9	113.8 (2)	C24—C25—C26A	108.7 (6)
C12—C11—H11A	108.8	C27—C25—C26A	102.1 (6)
C9—C11—H11A	108.8	C26B—C25—C24	124.7 (6)
C12—C11—H11B	108.8	C26B—C25—C27	123.3 (6)
C9—C11—H11B	108.8	C24—C25—C27	111.1 (3)
H11A—C11—H11B	107.7	C24—C25—H25A	111.5
C13—C12—C11	112.2 (2)	C27—C25—H25A	111.5
C13—C12—H12A	109.2	C24—C25—H25B	93.1
C11—C12—H12A	109.2	C27—C25—H25B	93.1
C13—C12—H12B	109.2	O26A—C26A—C25	115.4 (8)
C11—C12—H12B	109.2	O26A—C26A—H26A	108.4
H12A—C12—H12B	107.9	O26A—C26A—H26B	108.4
C12—C13—C14	107.25 (19)	C25—C26A—H26A	108.4
C12—C13—C17	115.32 (19)	C25—C26A—H26B	108.4
C14—C13—C17	99.9 (2)	H26A—C26A—H26B	107.5
C12—C13—C18	109.8 (2)	C26A—O26A—H26E	109.5
C14—C13—C18	112.3 (2)	C25—C26B—H26C	113.2
C17—C13—C18	111.84 (19)	C25—C26B—H26D	113.2
C15—C14—C8	120.2 (2)	H26C—C26B—H26D	110.5
C15—C14—C13	103.86 (18)	C26B—O26B—H26F	109.5
C8—C14—C13	115.1 (2)	C25—C26B—O26B	92.8 (8)
C15—C14—H14A	105.5	O26B—C26B—H26C	113.1
C8—C14—H14A	105.5	O26B—C26B—H26D	113.2
C13—C14—H14A	105.5	C25—C27—H27A	109.5
C14—C15—C16	102.9 (2)	C25—C27—H27B	109.5
C14—C15—H15A	111.2	C25—C27—H27C	109.5
C16—C15—H15A	111.2	H27A—C27—H27B	109.5
C14—C15—H15B	111.2	H27A—C27—H27C	109.5
C16—C15—H15B	111.2	H27B—C27—H27C	109.5
			
C10—C1—C2—C3	56.9 (3)	C18—C13—C14—C8	61.4 (3)
C1—C2—C3—O3'	70.4 (3)	C8—C14—C15—C16	−168.6 (2)
C1—C2—C3—C4	−52.4 (3)	C13—C14—C15—C16	−38.0 (3)
O3'—C3—C4—C5	−67.8 (3)	C14—C15—C16—O22'	129.1 (2)
C2—C3—C4—C5	51.9 (3)	C14—C15—C16—C17	14.4 (3)
C3—C4—C5—C6	−179.7 (2)	O22'—C16—C17—C20	22.5 (2)
C3—C4—C5—C10	−53.5 (3)	C15—C16—C17—C20	141.2 (2)
C4—C5—C6—C7	70.7 (3)	O22'—C16—C17—C13	−104.4 (2)
C10—C5—C6—C7	−55.8 (3)	C15—C16—C17—C13	14.3 (2)
C5—C6—C7—C8	54.0 (3)	C12—C13—C17—C16	−151.2 (2)
C6—C7—C8—C14	−173.6 (2)	C14—C13—C17—C16	−36.6 (2)
C6—C7—C8—C9	−52.3 (3)	C18—C13—C17—C16	82.4 (2)
C14—C8—C9—C11	−53.3 (3)	C12—C13—C17—C20	93.7 (3)
C7—C8—C9—C11	−176.6 (2)	C14—C13—C17—C20	−151.8 (2)
C14—C8—C9—C10	176.6 (2)	C18—C13—C17—C20	−32.7 (3)
C7—C8—C9—C10	53.3 (3)	C16—C17—C20—C22	−25.8 (2)
C2—C1—C10—C19	−172.6 (2)	C13—C17—C20—C22	90.0 (3)
C2—C1—C10—C5	−54.8 (3)	C16—C17—C20—C21	93.3 (3)
C2—C1—C10—C9	65.9 (3)	C13—C17—C20—C21	−150.9 (2)
C6—C5—C10—C1	177.4 (2)	C21—C20—C22—O22'	−98.0 (3)
C4—C5—C10—C1	51.7 (3)	C17—C20—C22—O22'	21.0 (3)
C6—C5—C10—C19	−67.0 (3)	C21—C20—C22—C23	84.6 (4)
C4—C5—C10—C19	167.4 (2)	C17—C20—C22—C23	−156.4 (3)
C6—C5—C10—C9	54.8 (3)	C23—C22—O22'—C16	170.9 (2)
C4—C5—C10—C9	−70.9 (3)	C20—C22—O22'—C16	−7.0 (3)
C11—C9—C10—C1	58.2 (3)	C15—C16—O22'—C22	−126.5 (2)
C8—C9—C10—C1	−173.4 (2)	C17—C16—O22'—C22	−10.3 (3)
C11—C9—C10—C19	−61.0 (3)	C20—C22—C23—C23'	−3.5 (4)
C8—C9—C10—C19	67.4 (3)	C20—C22—C23—C24	175.4 (3)
C11—C9—C10—C5	177.7 (2)	O22'—C22—C23—C23'	179.3 (2)
C8—C9—C10—C5	−53.9 (2)	O22'—C22—C23—C24	−1.8 (4)
C8—C9—C11—C12	52.6 (3)	C22—C23—C23'—O23"	−12.3 (4)
C10—C9—C11—C12	−178.2 (2)	C24—C23—C23'—O23"	168.8 (3)
C9—C11—C12—C13	−53.4 (3)	C22—C23—C23'—C23"	167.7 (3)
C11—C12—C13—C14	53.4 (3)	C24—C23—C23'—C23"	−11.2 (4)
C11—C12—C13—C17	163.7 (2)	C22—C23—C24—C25	79.4 (4)
C11—C12—C13—C18	−68.9 (3)	C23'—C23—C24—C25	−101.7 (4)
C7—C8—C14—C15	−51.6 (3)	C23—C24—C25—C26B	22.1 (8)
C9—C8—C14—C15	−175.1 (2)	C23—C24—C25—C27	−168.3 (3)
C7—C8—C14—C13	−177.1 (2)	C23—C24—C25—C26A	80.1 (7)
C9—C8—C14—C13	59.5 (2)	C26B—C25—C26A—O26A	50.6 (11)
C12—C13—C14—C15	167.2 (2)	C24—C25—C26A—O26A	−69.2 (12)
C17—C13—C14—C15	46.6 (2)	C27—C25—C26A—O26A	173.3 (9)
C18—C13—C14—C15	−72.1 (3)	C24—C25—C26B—O26B	85.8 (11)
C12—C13—C14—C8	−59.3 (3)	C27—C25—C26B—O26B	−82.5 (11)
C17—C13—C14—C8	−179.86 (19)	C26A—C25—C26B—O26B	−2.6 (12)

### Hydrogen-bond geometry (Å, °)

**Table:**

*D*—H···*A*	*D*—H	H···*A*	*D*···*A*	*D*—H···*A*
O3'—H3'···O23"^i^	0.98	1.89	2.841 (3)	163
O26A—H26E···O3'^ii^	0.82	2.20	2.951 (6)	153
O26B—H26F···O3'^ii^	0.82	2.24	2.919 (16)	140

Symmetry codes: (i) *x*, *y*, *z*−1; (ii) *x*, *y*+1, *z*+1.
